# Certified Versus Pasteurized Milk

**Published:** 1914-06

**Authors:** C. W. Gould


Candler Bldg., Atlanta, Ga.

---

### CERTIFIED VERSUS PASTEURIZED MILK.

By C. W. Gould M. D.

Because all forms of micro-organisms find such a favorable habitat in milk, it is the cause of more sickness and deaths than all other foods combined. Also it is the most easily decomposed, therefore hard to handle in a fresh and clean condition.

Typhoid epidemics, innumerable, have been traced to milk drawn by Typhoid Bacillus carriers. A Washington, D. C., epidemic was traced to a woman who washed the milk cans for her husband's dairy. What avails clean stables, with the windows screened, the cans washed, the miklers wearing white aseptic uniforms and having sterile hands, if there is one "Typhoid Carrier" in the chain of people from the bottle washers down to the last one who does anything to the bottle before it is finally sealed? Grave Tonsilitis epidemics are milk borne, as Capps and Rosenow of Chicago found. So if one of the milkers cough ever so discreetly and he happens to be a Streptococcus or Diphtheria Bacillus carrier he may be the cause of much suffering and many deaths! If a milk handler is a Tuberculous subject, milk may easily become contaminated with the Tubercle Bacillus of the human type, and, of course, even a tuberculin tested cow might have Tuberculosis in the early stages without betraying the fact when she was tested. The Tubercle Bacillus is not betrayed by the bacteriological examination of milk, possible where so many specimens are being examined in the city laboratories. Ordinary cultural methods do not bring them out.

Dr. E. C. Rosenow, of the Memorial Institute for Infectious Diseases of Chicago, carried out a line of experiments which he published in the Journal of Infectious Diseases, wherein he proved that a cow's udder may contain Hemolitic Streptococci and Pneumococci capable of producing Endocarditis, Arthritis and Pneumonia in laboratory animals. He did this by going to a dairy which is considered one of the best in his community and which supplies certified milk to its customers, and from normal healthy cows, he drew by aseptic methods through a sterile catheter, the milk into the culture media. He also cultured the centrifugalized sediment from cream separators and found virulent pathogenic organisms in that material.

In passing, it would seem that for those who prefer raw certified milk and are able to pay for it, that not merely the number of bacteria should be determined, but the kind, before the milk is recommended as fit for human consumption in the raw state. As Pathogenic Streptococci and Pneumococci are revealed quickly in blood agar at a glance, and the use of Endo

Media or Hess Stokes Semi-solid media quickly shows the Colon and Typhoid groups, the writer would respectfully suggest the use of such special media in the examination of Certified milk. In fact, because of the possibility of pathogenic organisms being introduced in the milk handled in the most careful manner, certification is of little value, with the exception of lessening the liability to a great extent of such contamination.

The fact that the milk of a dairy should be tried for pathogenic organisms is not only fully established but in addition to that, after the demonstration is made that such organisms are found in the milk, each cow's milk of that herd should be examined separately until the animal or animals contributing these organisms may be isolated from the other cows. In that way the farmer will be saved a great hardship.

Garget in a cow's udder has been proven to be the source of organisms which have been the cause of epidemics of sore throats and other infections of consumers.

If the Typhoid Bacillus has been isolated from the milk of a dairy, the carrier should be sought for. There is always one in such a case.

However, the above remarks are aside from the subject. The point is, we want to eliminate all pathogenic organisms. To do this there is only one way, that is by heat of the milk. If milk is heated in the open and at too high a temperature, the cream line is destroyed by changing the globules of fat. If milk is boiled the following pronounced changes take place, according to M. J. Rosenau of Harvard, "partial decomposition of proteids and other complex nitrogenous derivatives, diminution of the organic phosphorus; and an increase of inorganic phosphorus; precipitation of calcium and magnesium salts, and the greater part of the phosphates, expulsion of the greater part of the carbon dioxide, caramelization or burning of a certain portion of the milk sugar, causing the brownish color; partial derangement of the normal emulsion and coalesence of some of the fat globules, coagulation of the serum albumin, which begins at 75 degrees C; the ferments are killed."

Boiled milk has a cooked taste which appears at about 70 degrees C. This is due perhaps to the decomposition of certain gases and also influences the taste, so milk heated in closed vessels has a less pronounced flavor than if heated in open vessels. If heated in the open air a pellicle forms, which renews if it is removed. This scum forms at about 60 degrees C. and consists of fatty matter 45.42%, Casein and albuminoids 50.86%, Ash 3.72%. Heated in the closed vessels the pellicle does not form. It seems this pellicle is due to the drying of the upper layer of the liquid. It has been observed that cooked milk coagulates with rennin more slowly than does raw milk. The effect is noted at temperatures of 80 or 90 degrees C. but not in milk heated to 60 degrees C. for 20 minutes. However, the rennin of cooked milk is softer, less tough and more floculent tha n that produced by rennin coagulation of raw milk. Therefore cooked milk is more easily digested.

As heating milk at 6o degrees C. for 20 minutes kills pathogenic Streptococci, Staphylococci, Typhoid, Colon, Diphtheria and Tubercle bacilli, the organisms of scarlet fever, malta fever, dysentery and foot-and-mouth disease, in fact, about all the non spore-bearing organisms pathogenic to man, milk should be pasteurized by the City after being bottled so as not to leave it to individual caprice. After heating, the milk should be rapidly cooled. This method would cost little and save many lives. Of course, people who can afford to pay the high prices demanded for certified milk could have it, but even then they would not be sure that the organisms present were not pathogenic, while the methods used by most cities at present obtain.

Pasteurization should not be allowed to be an excuse for gross carelessness on the part of the producers. All should be held to a reasonable standard so that ilk would not contain harmful bacteriological toxins, which would not be destroyed by heat. This would give the children of the poor a chance to live as well as the more fortunate children of the well-to-do.

Candler Bldg., Atlanta, Ga.

### REGULAR MEETING FULTON COUNTY MEDICAL SOCIETY.

By R. R. Daly, M. D.

Dr. C. W. Gould read a paper on "Pasteurized versus Certified Milk."

Dr. Claude Smith said that Pasteurization had been experimented with for many years and that Strauss of New York had caused great advances to be made in the process. The difficulty has been to regulate the temeprature to exactly 165 F. and to keep the milk under proper and safe conditions after this process has been applied. Cooking milk devitalizes it in some respects. The Germans have showed that the "live" qualities of all foods are desirable when they can be used. Pasteurization does kill off certain good things in the milk, but that loss is more than offset by the safety of the sterile milk when the work is properly done. Certified milk means clean milk and that in turn, means clean dairies and workmen as well as healthy cattle. It involves a sterilizing plant for cans and other containers so that live steam at at least 10 lbs. pressure can be applied to the utensils for an hour. It necessitates also a corps of inspectors to see that the work is well done and to give instruction whenever needed. This costs money and the people must pay for it if they expect to get this kind of safety. Pasteurized milk seems to be returning to favor recently because of the better methods of controlling the heat that have been devised.

Dr. C. A. Rhodes said that there was no comparison between the two milks when it came to feeding children. Certified milk was the only thing to use when possible to secure it. But certified milk for a whole city at this time was impossible because people could not be educated and trained to produce it. Pasteurized milk was the recourse for adults and the poor. However, any milk that was dirty to begin with could not be freed from its toxins by Pasteurization. The chemic elements were there and the temperature of 165 F. did not change them. It stood every community in hand to have as clean milk as possible all the time in advance of Pasteurization.

Dr. J. C. Olmsted said that in his experience there had been some occasion to fear Ricketts as a result of Pasteurized milk.

Dr. J. W. Duncan said that if the temperature arose high enough to cause boiling or cooking, the milk was not well digested by babies.

Dr. Gould, in closing, said that the food values of boiled milk are not so great as those of the fresh samples but the caseins were broken up and, as a matter of fact, were more easily digested.

Dr. W. E. Person read a paper "Fractures of the Lower End of the Humerus, Involving the Elbow Joint."

Dr. J. L. Campbell said that inasmuch as we all have to attend fractures in emergencies at times, this paper was of importance. The point of spreading of the bones in "T" fractures was well made and the condition should be remembered. The X-ray after the plasterof Paris had been applied was of value and should be used whenever possible. He believes that the carrying angle should be carefully considered whenever there is the least danger of subsequent ankylosis and this always was a danger when the joint was involved. Dressings should never be so tight as to make the patient uncomfortable.

Dr. F. K. Boland read a paper "Injuries of the Kidney."

Dr. W. A. Selman said that he had a case of rupture of the kidney at the same time with those reported. The man came late to Grady Hospital. He had blood in the urine, increasing tumor in the right illiac region and some pain. Operation showed stellate rupture of the kidney. Recovery.

Dr.. J. R. McCord said that his brother had fallen from a tree and had much blood in urine for several days. In two weeks' time he recovered without treatment.

Dr. E. P. Merritt said that washing the bladder with warm saline would permit cystoscopy and determine which side the blood came from or if it had some other than a renal source.

Dr. J. L. Campbell said that while he had had no experience with rupture of the kidney he had had secondary haemorrhage into the bladder from other causes. Cystotomy had been necessary to remove painful clots. In another case Cyanide solution 1—5000 had washed the clots away.

Dr. W. E. Person said that he would be afraid to use cyanide in the bladder in the way suggested, and that he believed a two-way catheter with plenty of saline would accomplish the cleansing. The introduction of a sterile catheter should be made early for the purpose of diagnosis.

Dr. Boland reiterated his two points: Great violence to the kidney without external wound and constancy of line of tear in the kidney. These constituted a definite picture of the trouble.

Dr. R. R. Daly related a case of Partial Paresis of the Left Vocal Cord together with ulcer of the inner arytenoid space and also of the trachea. It was not specific or malignant but had been overlooked. The man could hardly speak. He was directed not to use his voice above a whisper. Appropriate local applications were made together with massage and large doses of strychnine and the man recovered in 20 days after having suffered for three months.

---

### THE WELLCOME HISTORICAL MEDICAL MUSEUM.

The Historical Medical Museum, which was founded by Mr. Henry S. Wellcome, in connection with the Seventeenth International Congress of Medicine, was reopened on May 28th as a permanent institution in London. It is now known as the "Wellcome Historical Medical Museum" and is open daily from 10 a. m. to 6 p. m., closing at 1 p. m. on Saturday; entrance 54-A Wigmore Street, Cavendish Square, W. Since closing last October the collections in the Museum have been considerably augmented and entirely rearranged. Many objects of importance and interest have been added, which it is hoped will increase the usefulness of the Museum to those interested in the History of Medicine. Members of the medical and kindred profession are admitted on presenting their visiting cards. Tickets of admission may be obtained by others interested in the History of Medicine on application to the Curator, accompanied by an introduction from a registered medical practitioner. Ladies will be admitted, only if accompanied by a qualified medical man.

